# Hypertrophic cardiomyopathy in a 20-year-old woman

**DOI:** 10.1007/s12471-015-0662-0

**Published:** 2015-02-26

**Authors:** E.O.F. van Gorselen, R. Schuurman, W. Terpstra

**Affiliations:** 1 Department of Cardiology, Slingeland Hospital, Kruisbergseweg 25, 7000 AD Doetinchem, The Netherlands; 2 Department of Cardiology, Medisch Spectrum Twente, Haaksbergerstraat 55, 7513 ER Enschede, The Netherlands

**Keywords:** Hypertrophic cardiomyopathy, Echocardiography, Cardiac magnetic resonance imaging, Cardiac surgery, ICD therapy, Genetics

## Abstract

A case of a young woman with complaints of shortness of breath and recurrent collapses is presented, including echocardiographic and cardiac MRI images showing extremely hypertrophied myocardium due to hypertrophic cardiomyopathy. The patient was referred for therapy and genetic counselling.

A 20-year-old woman was seen with palpitations, collapses and shortness of breath. Her father died at the age of 33 years. Echocardiography showed an extremely hypertrophied interventricular septum (Fig. 1a–d). The typical dagger shape seen upon Doppler imaging displays a maximum pressure gradient of 41 mmHg at rest. A septal thickness of 42 mm was measured on cardiac magnetic resonance imaging (Fig. 1e–h). No systolic anterior movement of the mitral valve was appreciated. Late gadolinium enhancement showed mild intramural contrast enhancement of the basal septal region. Holter registration showed periods of non-sustained ventricular tachycardia. The patient underwent implantable cardioverter-defibrillator placement and septal myectomy [1]. At genetic counselling a mutation was found in the MYBPC3 gene. Hypertrophic cardiomyopathy is the most common hereditable cardiovascular disorder with an estimated prevalence of 0.2 % [2]. The pattern of inheritance is autosomal dominant. It is the most common cause of sudden cardiac death in young individuals.

**Fig. 1:**
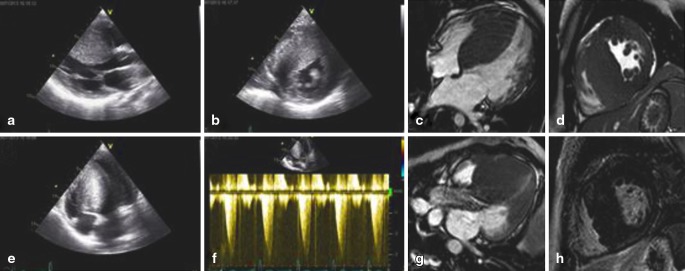
Echocardiography: parasternal long axis (**a**), parasternal short axis (**b**), apical 4-chamber view (**c**) and continuous wave Doppler of the outflow tract region (**d**), Cardiac MRI: cine image horizontal long axis view (**e**), short axis view (**f**), LVOT view (**g**) and LGE short axis view (**h**)
